# Carrier-assisted differential detection

**DOI:** 10.1038/s41377-020-0253-8

**Published:** 2020-02-10

**Authors:** William Shieh, Chuanbowen Sun, Honglin Ji

**Affiliations:** Department of Electrical and Electronic Engineering, The University of Melbourne, Parkville, VIC3010 Australia

**Keywords:** Optical techniques, Applied optics

## Abstract

To overcome power fading induced by chromatic dispersion in optical fiber communications, optical field recovery is a promising solution for direct detection short-reach applications, such as fast-evolving data center interconnects (DCIs). To date, various direct detection schemes capable of optical field recovery have been proposed, including Kramers−Kronig (KK) and signal−signal beat interference (SSBI) iterative cancellation (IC) receivers. However, they are all restricted to the single sideband (SSB) modulation format, thus conspicuously losing half of the electrical spectral efficiency (SE) compared with double sideband (DSB) modulation. Additionally, SSB suffers from the noise folding issue, requiring a precise optical filter that complicates the receiver design. As such, it is highly desirable to investigate the field recovery of DSB signals via direct detection. In this paper, for the first time, we propose a novel receiver scheme called carrier-assisted differential detection (CADD) to realize optical field recovery of complex-valued DSB signals via direct detection. First, CADD doubles the electrical SE compared with the KK and SSBI IC receivers by adopting DSB modulation without sacrificing receiver sensitivities. Furthermore, by using direct detection without needing a precise receiver optical filter, CADD can employ cost-effective uncooled lasers as opposed to expensive temperature-controlled lasers in coherent systems. Our proposed receiver architecture opens a new class of direct detection schemes that are suitable for photonic integration analogous to homodyne receivers in coherent detection.

## Making direct detection more coherent

A novel receiver decodes fiber optic “double sideband signals” with high efficiency, using a method called direct detection, which is cheaper than the more complex coherent detection used in long-distance data transport. The receiver, developed by William Shieh and colleagues at the University of Melbourne, Australia, recovers intensity and phase data from the signals and almost doubles the electrical spectrum efficiency compared to some currently proposed receivers. Called carrier-assisted differential detection (CADD), the receiver suppresses a type of interference called signal−signal beating interference, and can use cost-effective uncooled lasers, without a need for a precise receiver optical filter. CADD could provide a compact, cost-effective solution for short-distance communications, such as in data center interconnects.

## Introduction

Coherent detection has profoundly impacted optical communications due to its superior capability of recovering both optical intensity and phase, namely, field recovery^1,2^. Distinct from conventional intensity modulation with direct detection, field recovery enables in-phase/quadrature (IQ) modulation, increasing the transmission spectral efficiency (SE). Moreover, optical field impairments such as chromatic dispersion and polarization mode dispersion can be digitally compensated by accessing the field information^3–5^. However, coherent transceivers are relatively costly due to the hardware complexity and tight specifications for lasers^6^. To address hardware complexity, photonic integration has become a promising solution^7^, but the issue of precise frequency control between the local oscillator and transmitter laser is inevitable. Consequently, coherent detection remains a suitable solution for medium- to long-haul transport^8^, while direct detection is still dominant for short-reach applications, such as data center interconnects^9,10^. For direct detection, loss of field recovery is the main obstacle to digitally compensating chromatic dispersion, limiting the transmission reach for conventional IM/DD systems^11^. To bridge the gap between direct and coherent detection, a self-coherent scheme has attracted extensive research interest, in which a strong carrier is inserted at the transmitter and propagated along with the information-bearing signals. After square-law detection using a single-ended photodiode (PD), signals can be extracted from the signal-carrier beating term, and the optical field is reconstructed without using a local oscillator. In the recent decade, various schemes of field recovery with direct detection have been investigated^12–23^. Since direct detection generally provides only intensity information, until now, signals have been mainly restricted to the single sideband (SSB) modulation format in various proposed intensity-only detection schemes^14^. For such detection schemes, signal−signal beating interference (SSBI) is the dominant limitation. To mitigate SSBI, a frequency gap, which is commonly as wide as the signal bandwidth, can be placed between the carrier and signals^15^. To overcome the poor SE of the above approach, a self-coherent scheme without a frequency gap has been proposed in which SSBI can be estimated and then subtracted in an iterative manner^16–18^. In recent years, the Kramers−Kronig (KK) receiver has been proposed to effectively mitigate SSBI without using iterations^19^. Via KK relations, the phase of signals is obtained using the intensity information. Since the SSB modulation format is adopted for KK receivers, twin-SSB^20,21^ and WDM^22,23^-based KK receivers implemented with optical filters have been proposed to fully utilize the optical spectrum. Compared to the optical SE, however, a high electrical SE is a more dictating factor for short-reach applications. For KK or SSBI iterative cancellation receivers, the electrical SE is intrinsically limited by the SSB modulation format. Since one sideband is unfilled, half of the electrical SE is lost. Apart from the electrical SE, SSB signals suffer from noise folding due to the square-law detection of the photodiode. Consequently, rather than SSB signals, it is highly desirable to investigate the direct detection of complex-valued double sideband (DSB) signals with field recovery. Although there are some demonstrations of DSB direct detection via block-wise phase switching^24,25^, the effective SE is reduced by half due to the repetition of data.

In this paper, we propose a novel receiver scheme for detecting complex-valued double sideband (DSB) signals with field recovery, called carrier-assisted differential detection (CADD). Both the lower and upper sidebands are filled with uncorrelated information-bearing signals. Compared with SSB modulation, the electrical SE is increased by a factor of approximately 1.8 without sacrificing the receiver sensitivity. In addition, no precise optical filters are needed for the CADD receiver, indicating the potential of utilizing low-cost uncooled lasers for the CADD receiver scheme. CADD possesses two advantages over conventional carrier-less differential detection (CDD)^5^ for field recovery: (i) CADD doubles the electrical SE compared to CDD, as CADD recovers the linear signal while CDD needs to recover the second-order signal-to-signal beating term, and (ii) CADD is insensitive to chromatic dispersion, while CDD is not. This is because without a carrier, the field of CDD can reach zero, which makes differential detection impossible for large chromatic dispersion. The advantage of CADD over the KK receiver in direct detection is analogous to that of homodyne over heterodyne receivers in coherent detection—although CADD requires a larger number of components, it reduces the optoelectronic bandwidth by half. By adopting photonic integration, either in the InP or silicon photonics (SiP) platform, the large component count in CADD will be much mitigated, while the reduced bandwidth of CADD will greatly reduce the overall implementation cost. Compared to coherent homodyne receivers, CADD does not require highly stable and low-linewidth lasers, leading to a more compact and cost-effective solution suitable for short-reach applications such as intra-data interconnects and ultra-high-speed wireless fronthaul networks.

## Results

### Architecture of the CADD receiver

To reconstruct the optical field, a carrier is necessary to obtain the desired carrier-signal beating term. We denote the carrier and signal field as *C* and *S*, respectively. Assuming that the responsivity of the photodiode equals 1 for simplicity, after square-law detection, the received photocurrent *I* can be expressed as

1 $$I = \left| {C + S} \right|^2 \,=\, \left| C \right|^2 \,+ \,\left| S \right|^2 \,+ \, 2{\mathrm{Re}} [C^ \ast \cdot S]$$

where * stands for conjugation, and Re[·] stands for the real part. For the right-hand side of the above equation, only the last term 2Re[C*·*S*] is the desired term. Since this term represents the real value, SSB signals with real and imaginary parts satisfying the Hilbert transform can be recovered, while complex-valued DSB signals with no such property cannot be recovered merely via the term 2Re[C*·*S*].

**Fig. 1:**
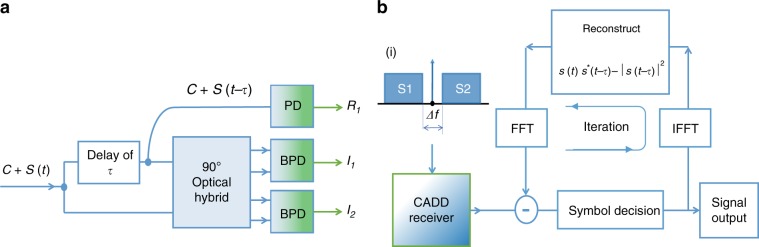
**a** Receiver scheme for CADD; **b** DSP for OFDM modulated signals using the CADD receiver. Inset (i) is the spectrum of signals fed to the CADD receiver, where S1 and S2 are lower and upper sideband signals, respectively. *PD* photodiode, *BPD* balanced photodiode, *FFT* fast Fourier transform, *IFFT* inverse fast Fourier transform

Figure 1a depicts the structure for the proposed CADD receiver to recover complex-valued DSB signals. The input of the CADD receiver consists of the carrier and the signals, denoted by *C* *+* *S*(*t*). An optical coupler is utilized to split the input into two paths, with an optical delay of time *τ* on one path, corresponding to *C* + *S*(*t* − *τ*). Without loss of generality, we have assumed that the carrier *C* is a real-valued constant. This is justified, as the optical delay *τ* is on the order of the baud period, and the carrier phase change would be insignificant with a delay of *τ*. The delayed path is further split into two branches, one of which is fed into a single-ended photodiode. The photocurrent of the single-ended photodiode *R*_1_ is expressed as

2 $$\begin{array}{l}R_1 = \left| {C + S(t - \tau )} \right|^2 \,=\, \left| C \right|^2 \,+\, \left| {S(t - \tau )} \right|^2\\ + \,C[S(t - \tau ) + S^ \ast (t - \tau )]\end{array}$$

The two optical signals at the output of the coupler, *C* + *S*(*t*) and *C* + *S*(*t* – *τ*), are input into an optical hybrid and then fed into two balanced photodiodes (BPDs). The photocurrents of the two BPDs, *I*_1_ and *I*_2_, are thus given by

3 $$\begin{array}{l}I_1 = 4{\mathrm{Re}} \{ [C + S(t - \tau )]^ \ast \cdot [C + S(t)]\} I_2\\ = 4{\mathrm{Im}} \{ [C + S(t - \tau )]^ \ast \cdot [C + S(t)]\} \end{array}$$

where Re{·} and Im{·} represent the real and imaginary parts, respectively. It is worth noting that a 3 × 3 coupler can serve the same function as the 90° optical hybrid^26^ with a lower cost. We reconstruct a complex-valued signal from *I*_1_ and *I*_2_ as

4 $$\begin{array}{l}R_2 = (I_1 + jI_2)/4 = \left| C \right|^2 \,+\, C[S(t) + S^ \ast (t - \tau )]\\ + S(t) \cdot S^ \ast (t - \tau )\end{array}$$

Strictly, *C* and *S* should be expressed as $Ce^{j2\pi f_0t}$ and $Se^{j2\pi f_0t}$, where *f*_0_ is the carrier frequency. As such, there exists an additional common phase term in *R*_2_, which can be easily estimated and compensated with receiver digital signal processing (DSP). We subtract *R*_1_ from *R*_2_ and obtain

5 $$R = R_2 - R_1 = C[S(t) - S(t - \tau )] + S_2$$

where $S_2 = S(t)S^ \ast (t - \tau ) - \left| {S(t - \tau )} \right|^2$, which is the second-order SSBI. It follows from Eq. (5) that the desired linear term *S*(*t*) − *S*(*t* − *τ*) can be expressed as

6 $$S(t) - S(t - \tau ) = \left( {R - S_2} \right){\mathrm{/}}C$$

Taking the Fourier transform of Eq. (6), we obtain

7 $$\begin{array}{ccccc}\\ S(f) = \left( {1 - e^{j2\pi f\tau }} \right)^{ - 1}{\cal{F}}\{ \left( {R - S_2} \right)/C\} \cr \\ = H(f)^{ - 1}{\cal{F}}\{ \left( {R - S_2} \right)/C\} \\ \end{array}$$

where *S*(*f*) is the Fourier transform of *S*(*t*), namely, $S(f) = {\mathcal{F}}\{ S(t)\}$. We define the transfer function of the CADD receiver as *H*(*f*), which equals 1 − *e*^*j*2*πfτ*^, in essence, the transfer function of an interferometer with a delay of *τ*. As shown in Eq. (5), despite SSBI distortions *S*_2_, the desired term of $S(t) - S(t - \tau )$ is amplified by the carrier *C*. Thus, a strong carrier mitigates the effects of SSBI distortions, which in turn enables relatively accurate preliminary symbol decisions using *R*. Equation (7) is the main formula used to reconstruct *S*(*f*). The SSBI term *S*_2_ can be reconstructed via the preliminary symbol decision and then subtracted from *R* iteratively. The preliminary symbol decision is made by setting *S*_2_ to zero. Further discussion on the transfer function *H*(*f*) can be found in the “Materials and methods” section.

Although the CADD architecture shown in Fig. 1a comprises three photodetectors, the required bandwidth of each one is reduced by half compared to SSB signal detection. For photonics integrated circuits (PICs), such as silicon photonics, the cost of an integrated circuit is mainly dominated by the electrical bandwidth of the circuit; namely, adding two more photodetectors into the PIC would not significantly increase the cost but double the receiver bandwidth would. Additionally, the system performance analysis is based on the optical signal-to-noise ratio (OSNR) sensitivity, and as such, the impact of receiver passive component loss and noise are immaterial and are not considered in this paper.

### Digital signal processing for CADD

Orthogonal frequency division multiplexing (OFDM) modulation is adopted to demonstrate the CADD receiver scheme. To enhance the SE, no cyclic prefix is inserted. As shown on the right-hand side of Eq. (7), transfer function *H*(*f*) has a null point at *f* = 0, and SSBI can be dramatically amplified around zero frequency. Hence, a small frequency gap (e.g., 10% of the signal bandwidth) is inserted in the vicinity of zero frequency. It is noted that such a frequency gap is not implemented to fully accommodate SSBI. In practice, the frequency of the gap for CADD can be merely approximately 10% of the signal bandwidth. For example, the frequency gap Δ*f* can be as narrow as 2.5 GHz for 25-Gbaud signals. The spectrum of signals along with the carrier is shown in insert (i) of Fig. 1b.

As shown in Fig. 1b, DSB signals along with a carrier are fed into the CADD receiver, with the same structure as depicted in Fig. 1a, which outputs the OFDM signal *S*(*f*) using Eq. (7) in the frequency domain. To eliminate SSBI *S*_2_ in an iterative manner, preliminary symbol decisions are made in the frequency domain for OFDM signals. IFFT is utilized to transform symbol decisions into the time domain signal *S*(*t*), and then, SSBI is reconstructed by using the relation $S_2 = S(t)S^ \ast (t - \tau ) - \left| {S(t - \tau )} \right|^2$. Since the output of CADD *S*(*f*) is in the frequency domain, FFT is needed to transform SSBI to the frequency domain and then subtract it from *S*(*f*). After several iterations (e.g., four iterations), the system performance converges, indicating that the SSBI has been effectively mitigated.

### System impact of the transfer function

The gist of the CADD receiver is to eliminate SSBI. As shown in Eqs. (5)−(7), we estimate $S(t) - S(t - \tau )$ via *R* by assuming that SSBI *S*_2_ equals zero for the first iteration. Although SSBI can be reconstructed and then eliminated iteratively, it is highly preferable to suppress SSBI before iterative cancellation. For the CADD receiver, its unique transfer function (which will be discussed further in “Materials and methods”) suggests a tactful approach to suppress SSBI by inserting a small frequency gap (e.g., 10% of the signal bandwidth). The benefit of the frequency gap in CADD is twofold: (i) SSBI, which is generally more severe in the vicinity of the zero frequency region, does not totally overlap with the signal spectrum due to the gap and hence produces less distortion for information-bearing signals; (ii) when the magnitude of the transfer function *H*(*f*) is greater than 1, SSBI is suppressed via the transfer function of CADD. Since the transfer function of the CADD receiver *H*(*f*) is also a function of optical delay *τ*, a desired frequency region with suppressed SSBI can be obtained by adjusting the optical delay *τ*.

**Fig. 2:**
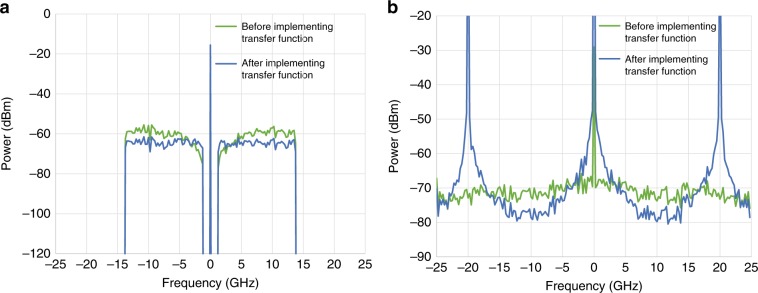
**a** Signal spectra before and after implementing transfer function *H*(*f*). **b** SSBI spectra before and after implementing transfer function *H*(*f*)

To demonstrate the effectiveness of SSBI suppression, we investigate the detection of 25-Gbaud 16QAM OFDM signals using the CADD receiver with a sampling rate of 50 Gsample/s. The optical delay is 50 ps, and the frequency gap is 2.5 GHz. The information-bearing signals occupy the bandwidths of [−13.75 GHz, −1.25 GHz] and [1.25 GHz, 13.75 GHz], indicating a frequency gap of 10% of the signal bandwidth. In Fig. 2a, the green dotted line represents the spectrum of *S*(*t*) − *S*(*t* − *τ*), with no distortions due to SSBI. After implementing transfer function *H*(*f*) as shown in Eq. (7), the spectrum of recovered signal *S*(*t*) is shown as the blue solid line in Fig. 2a. Spectra of SSBI are shown in Fig. 2b. Due to the transfer function of the CADD receiver, SSBI is significantly enhanced at frequencies of 0 and ±20 GHz. Since the null frequency of ±20 GHz is not within the information-bearing signal spectrum, these singularity spikes do not affect information-bearing signals. It is noted that in the frequency regions of [−16.7 GHz, −3.3 GHz] and [3.3 GHz, 16.7 GHz], SSBI can be suppressed by up to 6 dB. In addition, it can be concluded that SSBI suppression corresponds to an interplay between the frequency gap and optical delay, indicating that the CADD receiver can be optimized by inserting a frequency gap and tactfully adjusting the optical delay according to the signal bandwidth.

**Fig. 3:**
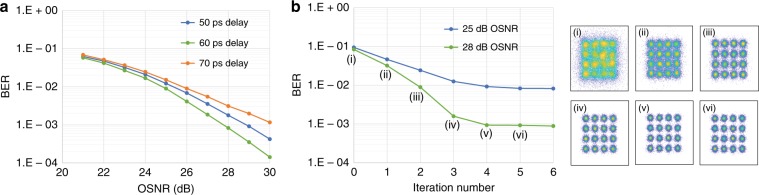
BER performance **a** versus OSNR for various delays and **b** versus the number of iterations @ a CSPR of 8 dB, an optical delay of 60 ps, and a frequency gap of 10%. Insets are the corresponding constellations for each iteration @ OSNR = 28 dB

To optimize the optical delay, we keep the frequency gap as a fixed value, which is 2.5 GHz for the 25-Gbaud signals. The carrier-to-signal power ratio (CSPR) is set to 8 dB, four iterations are implemented to cancel SSBI, and the BER as a function of the OSNR is shown in Fig. 3a. Since the carrier is transmitted along with signals, both the carrier and information-bearing signal power are considered as the “signal” power when calculating the OSNR. Among the various optical delay values shown in Fig. 3a, the delay of 60 ps is found to be the optimal.

The crux of optimizing the optical delay is to fit the information-bearing signals in the frequency region with SSBI suppression where the magnitude of the transfer function *H*(*f*) is greater than 1. When the delay is small, it is anticipated that the system performance will improve as the delay increases. This is the nature of differential detection, in which the signal difference from the interferometer is enhanced by using a larger delay. This is also manifested by the fact that the SSBI suppression region moves to a lower frequency when increasing the delay. However, when the interferometer delay becomes excessive, the second null point of the transfer function moves into the signal spectrum, degrading the system performance. As such, there exists an optimal delay for the receiver performance. To obtain the above results, we have used four iterations to mitigate SSBI. Figure 3b shows the BER as a function of iteration number. When the iteration number equals zero, we obtain the preliminary symbol decisions, which are used to reconstruct the SSBI, followed by iteration of SSBI mitigation. When the iteration number is greater than 4, more iterations do not bring about a substantial improvement. As such, in the following results, the iteration number is set to four.

### Carrier-to-signal power ratio of CADD

**Fig. 4:**
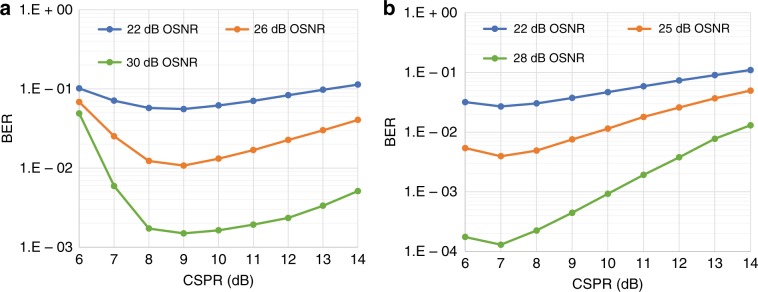
BER versus CSPR for 25-Gbaud signals **a** with a 5% frequency gap and **b** with a 20% frequency gap

In addition to the frequency gap and optical delay, the CSPR is another key factor to optimize for the CADD receiver. A high CSPR enlarges the desired term $S(t) - S(t - \tau )$ relative to the SSBI. However, this reduces the effective signal power due to the high carrier power and hence degrades the OSNR. Taking the CSPR into consideration, optimization of the CADD receiver is a three-parameter process involving varying the CSPR, frequency gap and optical delay. To maximize the electrical SE, it is preferable to narrow the frequency gap. For a given frequency gap, we sweep the CSPR from 6 to 14 dB to identify the optimal value. For the 25-GBaud DSB 16QAM signals, a 20% frequency gap indicates that the gap occupies 5 GHz [−2.5 GHz, 2.5 GHz], and signals occupy the frequencies of [−15 GHz, −2.5 GHz] and [2.5 GHz, 15 GHz]. Figure 4a, b depicts the BER as a function of CSPR for the 25-Gbaud 16QAM signals with frequency gaps of 5% and 20%, respectively.

For the 5% gap case, at the OSNR of 30 dB, the lowest BER occurs at a CSPR of 9 dB. As the OSNR decreases to 22 dB, the CSPR of 8 dB tends to have a similar performance as the 9-dB CSPR. For the 20% gap case, the optimal CSPR is 7 dB, as shown in Fig. 4b. This phenomenon indicates that the implementation of a frequency gap can relieve the requirement of high carrier power. This is because SSBI does not fully overlap with information-bearing signals, and a high CSPR is not required to obtain a strong replica of signals (e.g., $C[S(t) - S(t - \tau )]$ for CADD receivers).

### OSNR sensitivity

To demonstrate the OSNR sensitivity of the CADD receiver with various frequency gaps, we optimize the optical delay and CSPR as discussed above. The frequency gap ranges from 5 to 25% for 25-Gbaud signals. The optimized parameters are listed in Table 1. The step sizes of the optimized optical delay and CSPR are 10 ps and 1 dB, respectively.

**Table 1:** Optimal delay and CSPR for various frequency gaps

Frequency gap (%)	Optical delay (ps)	CSPR (dB)
5	60	9
10	60	8
15	60	8
20	50	7
25	50	7

**Fig. 5:**
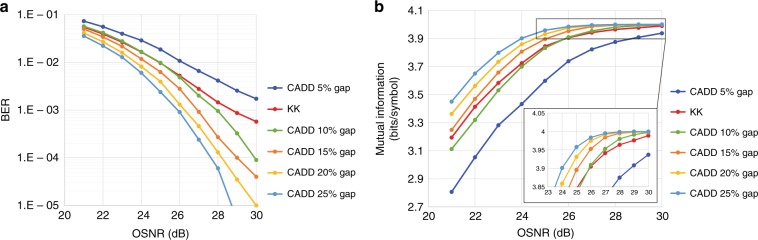
Back-to-back performance of the **a** OSNR sensitivity and **b** mutual information of CADD for various frequency gaps. The KK receiver is also included for reference

The optimal CSPRs shown in Table 1 generally decrease as the frequency gap becomes wider, which agrees with the analysis previously discussed. Meanwhile, the optical delay decreases from 60 to 50 ps, which is due to the interplay between the frequency gap and SSBI suppression. As we insert a wider gap in the low frequency region, information-bearing signals are pushed into the higher frequency region, and hence, the optical delay should be correspondingly decreased to guarantee that signals are in the SSBI suppressed region. We simulate the transmission of 25-Gbaud OFDM 16QAM DSB signals using our proposed CADD receiver scheme. As a representative SSB case for reference, the performance of the KK receiver is also presented. Considering the requirement of a high oversampling rate for KK receivers^27^, the sampling rate for the KK receiver is set to 100 Gsample/s, while for the CADD receiver, the sampling rate is 50 Gsample/s. With the same data rate of 100 Gbit/s, the OSNR sensitivities of the SSB case (e.g., KK receiver) and DSB case (e.g., CADD receiver) are presented in Fig. 5 in terms of the BER and mutual information (MI). For the CADD receiver, the OFDM modulation format is adopted. In contrast, a single carrier is adopted for the KK receiver due to the low peak-to-average power ratio (PAPR) of the single-carrier modulation format, which is beneficial for the KK receiver^28^, and no PAPR reduction technique is employed^29^. The CSPR of the KK receiver is 6 dB, which is the optimal value, and the corresponding optimal parameters for CADD are listed in Table 1. Aiming to avoid sophisticated wavelength stabilization and control, no optical filters are implemented for the KK and CADD receivers. Figure 5a shows that both the KK and CADD receivers are effective in mitigating SSBI. For the CADD receiver, even with a narrow frequency gap (e.g., merely 5%), the receiver algorithm still works properly. As the frequency gap increases, the CADD receiver can achieve better OSNR sensitivity with the adjustment of the optical delay and CSPR. At the BER threshold of 1 × 10^−3^, the OSNR sensitivity of the CADD receiver with a 10% gap is 28 dB. By inserting a wider frequency gap, the OSNR sensitivity of CADD is further improved. For example, without sacrificing much of the SE, the OSNR sensitivity of the CADD receiver with a 25% gap is approximately 26 dB. It can be concluded that for CADD receivers, there exists a trade-off between the SE and OSNR sensitivity. In addition to the BER, the mutual information (MI) of the CADD and KK receivers is depicted in Fig. 5b. Since the MI greatly converges when the OSNR is high, the inset of Fig. 5b displays the zoom-in detailed MI at high OSNRs.

**Fig. 6:**
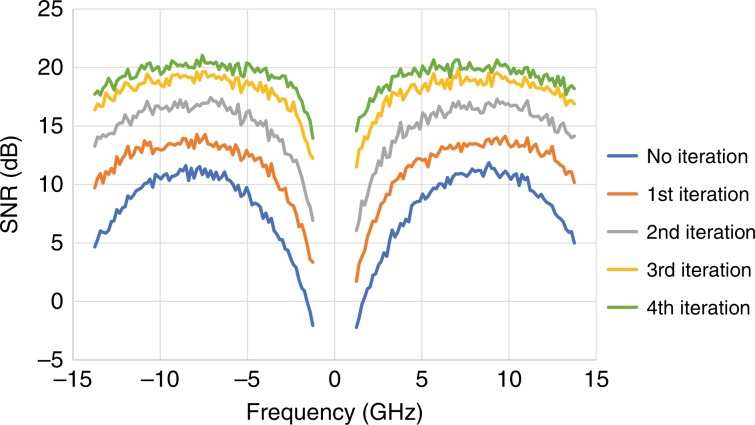
SNR versus frequency for 25-Gbaud signals with a 10% frequency gap, a 60-ps optical delay, an 8-dB CSPR, and a 30-dB OSNR

It is also worth noting that the transfer function of the CADD receiver is not uniform, leading to the signal-to-noise ratio (SNR) over the signal bandwidth not being uniform. Given that the OFDM modulation format is adopted for CADD receivers, we illustrate the SNR as a function of frequency for each iteration in Fig. 6. Since the frequency gap is 10%, the SNR is not displayed in the region of [−1.25 GHz, 1.25 GHz]. For the preliminary decision (e.g., no iteration is conducted), the SNR in the low frequency region is low, indicating that the SSBI in this region is severe. However, this is a colored-SNR channel; in the SSBI suppressed region, the SNR can be improved by more than 10 dB. After performing several iterations, the SNR gradually improves, and the characteristics of the colored SNR are mitigated. This is because for OFDM signals, SSBI is reconstructed in the time domain, while symbol decisions are made in the frequency domain, revealing the low correlation between the reconstructed SSBI and symbol decisions, and as such, SSBI can be sufficiently eliminated. This phenomenon fundamentally empowers effective SSBI mitigation for CADD receivers. After the fourth iteration, the average SNR is 19.7 dB, with the lowest SNR in the low frequency region of approximately 15 dB. We also observe that when inserting a wider frequency gap (e.g., a 20% gap), the SNR curve over the signal bandwidth is more uniform than that in the 10% gap case shown in Fig. 6.

## Discussion

After thoroughly studying the system performance of CADD, we are in a good position to reproduce the comparison between different coherent and direct detection schemes presented in ref. ^30^ using some relevant cost metrics, as shown in Table 2. For a fair comparison, we also assume that all the detection schemes aim to achieve 200 Gb/s per polarization per wavelength at an OSNR of 30 dB. In our view, the optoelectronic bandwidth and whether a coherent laser is required are the two most important contributing factors to the implementation cost of photonic integrated circuits. As shown in Table 2, coherent homodyne detection outperforms all the other modulation formats in the bandwidth requirement. Although it requires twice as many components as coherent heterodyne detection, the reduced electrical bandwidth for homodyne detection is such a predominant advantage that all the field-deployed coherent systems use the homodyne architecture. Similarly, in the direct detection domain, although CADD requires more components, due to the unique capability of detecting DSB signals, the required electrical bandwidth for CADD is reduced by almost half, and therefore, it is greatly positioned to be implemented in photonic integrated circuits. The advantage of CADD over the KK receiver is analogous to that of homodyne over heterodyne receivers in coherent detection. As such, we believe that our proposed receiver architecture opens a new class of direct detection schemes that are suitable for photonic integration analogous to homodyne receivers in coherent detection.

**Table 2:** Cost metrics of the 200-Gb/s net interface rate per wavelength per polarization detection system with field recovery

Modulation format	BW per ADC (GHz)	Requirement of stable lasers	Number of ADCs
Coherent (homodyne)	9.7	Yes	2
Coherent (heterodyne)	19.4	Yes	1
CADD^a^	16.0	No	3
KK^b^	31.6	No	1
Stokes^c^	25.1	No	3
Gapped SSB^d^	50.2	No	1
Interleaved SSB^e^	50.2	No	1

This table is reproduced from ref. ^30^ and the OSNR is set to 30 dB

^a^A 10% frequency gap is employed for the CADD receiver, with a CSPR of 8 dB

^b^The CSPR is 6 dB for the KK receiver^19^

^c^For the Stokes receiver^13^, modulated signals are in the X polarization, and the Y polarization is occupied by the carrier, with a CSPR of 0 dB. Since this comparison table is based on single polarization, while both polarizations are loaded with either signals or the carrier for the Stokes receiver, we include a multiplication factor of 2 for the bandwidth. In other words, all the bandwidths should be reduced by half when two polarizations are used to obtain the same net interface rate

^d^The frequency gap is as wide as the signal bandwidth, and the CSPR is 0 dB for the gapped SSB scheme^15^

^e^Odd-numbered subcarriers are loaded with signals, and even-numbered subcarriers are null. The CSPR is 0 dB for such an interleaved SSB scheme^31^

In summary, we have proposed a novel receiver scheme called CADD to recover the field of IQ modulated DSB signals via direct detection. CADD enables digital compensation of chromatic dispersion and almost doubles the electrical SE of KK or IC receivers. The transfer function of the CADD receiver is theoretically analyzed. It is shown that SSBI can be judiciously suppressed by taking advantage of the transfer function and further mitigated via iterative cancellation. Several key parameters, including the optical delay, frequency gap, and CSPR, are discussed and optimized. Additionally, the receiver sensitivity of CADD is presented, showing that the CADD receiver is robust to CD. This is the first realization of the field recovery of complex-valued DSB signals via direct detection with a low receiver bandwidth at almost half of the baud rate.

## Materials and methods

**Fig. 7:**
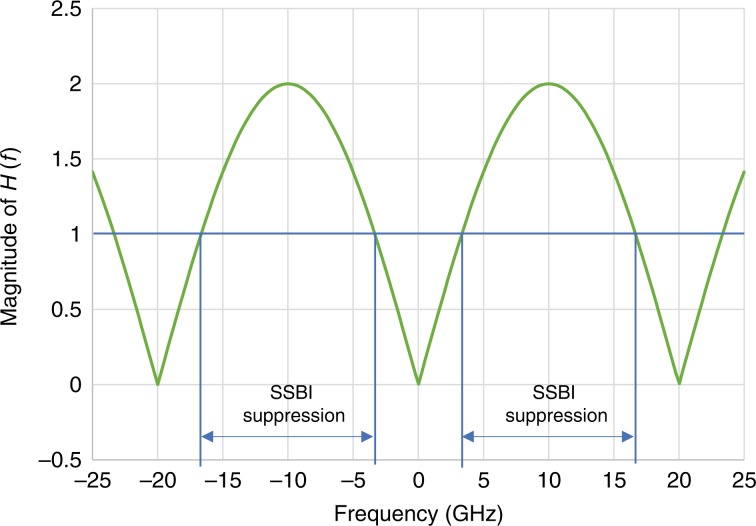
Magnitude of transfer function *H*(*f*) for CADD. SSBI suppression is defined as |*H*(*f*)| > 1

The unique and key characteristics of the CADD receiver lie in the transfer function *H*(*f*). It follows from Eq. (7), when *fτ* = 0, 1, 2, etc., that SSBI and noise can be severely enhanced when the transfer function *H*(*f*) approaches zero. An inserted frequency gap can prevent such distortion enhancement in the vicinity of zero frequency (e.g., *fτ* = *0*). However, the null point is inevitable for *fτ* = 0, 1, 2, etc. Hence, it is desirable to select an optical delay *τ* to avoid the null point being located inside the spectrum of the modulated signals. We set the optical delay *τ* to 50 ps and depict the transfer function *H*(*f*) in Fig. 7.

The magnitude of the transfer function equals zero at some specific frequencies. For example, at the frequencies of 0 and ±20 GHz, the magnitude of the transfer function is zero, leading to severe enhancement of SSBI distortions and noise. SSBI suppression is defined as |*H*(*f*)| > 1; for example, in the frequency regions of [−16.6 GHz, −3.4 GHz] and [3.4 GHz, 16.6 GHz], SSBI is suppressed.

To identify the system performance using the CADD receiver, we conduct a numerical simulation of a 25-Gbaud 16QAM signal using a commercial MATLAB program. Since the SNR is colored, OFDM signals are used for the simulation. The system parameters for OFDM are as follows: OFDM size of 1000 (in general, it should be a power of 2; here, we choose the subcarrier number of 1000 to facilitate the 5−25% frequency gap to obtain an integer number of percentage), subcarrier spacing of 25 MHz, and no use of a cyclic prefix. Before being fed into our proposed CADD receiver, additive white Gaussian noise is added to the signal to simulate optical noise. The bandwidth of both the single-ended and balanced photodetectors is set to 25 GHz, and the receiver sampling rate is 50 Gsample/s. For the BER computation, 320 OFDM symbols corresponding to 1,280,000 bits are collected with direct error counting.
